# Physicochemical Evaluation of Coated Ginger during Long-Term Storage: Impact of Chitosan and Beeswax Bilayer Coatings at Different Temperatures

**DOI:** 10.1155/2024/2054943

**Published:** 2024-05-31

**Authors:** Hernani, Christina Winarti, Tatang Hidayat, Abdullah Bin Arif, Sri Yuliani

**Affiliations:** Research Center for Agroindustry, National Research and Innovation Agency, Central Jakarta, Indonesia

## Abstract

Fresh ginger can spoil quickly owing to a variety of factors, including inappropriate postharvest handling, microbial and enzymatic activities, and chemical reactions during storage. This study evaluated the physicochemical properties of ginger coated with chitosan and beeswax during storage for 6 months at different temperatures (18°C and 25°C). Fresh ginger was treated with chitosan coating (1.5 and 3.5%), followed by beeswax coating (3 and 6%). The coated ginger was wrapped in a plastic net and stored at ambient (25°C) and low temperatures (18°C) for six months. The results confirmed that coating treatment slowed down the changes in physicochemical properties (moisture, phenolic content, and so on) of ginger during storage. Ginger stored at 25°C showed shorter shelf lives than those stored at 18°C. Coating ginger with 3% chitosan followed by 6% beeswax exhibited the best results in maintaining the moisture and phenolic content, reducing weight loss, and increasing total soluble solid (TSS) and cell compartment size for six months of storage. This study provides a promising approach to delaying the spoilage of fresh ginger by applying coating treatments useful for developing handling protocols for fresh ginger during storage and distribution.

## 1. Introduction

Ginger (*Zingiber officinale* Roscoe) is an essential medicinal and spice herb with many health benefits. It is widely used as a food ingredient, seasoning, supplement, and flavouring and has beneficial characteristics such as a spicy taste, aroma, nutrition, and pharmacological activity [1]. Due to its high moisture content and susceptibility to microbial attack, fresh ginger spoils quickly. Inappropriate storage conditions can reduce the quality of fresh ginger, characterised by drying loss, senescence, and putrefaction [2]. The primary causes of spoilage are improper postharvest handling, the growth of spoilage microbes, the action of naturally existing enzymes, chemical reactions, and structural changes during storage [3]. Fresh ginger is generally preserved using modified and controlled atmosphere packaging, chemical treatments such as colour fixing agents or inorganic salts, low temperature, and edible coatings, all of which are similarly used for fruit and vegetable preservation [4]. Furthermore, many eco-friendly postharvest treatments have been utilized for preservation of vegetables and fruits as well as stress reduction, including chitosan, oligochitosan [5], eugenol [6], humic acid, and methyl jasmonate [7, 8].

Chitosan is a linear polysaccharide made up of glucosamine and N-acetylglucosamine, connected by *β*-1,4-glycosidic linkages. It is obtained by partial deacetylation of chitin. The main distinction between chitin and chitosan is the proportion of acetyl groups in their chemical structures [9]. It is frequently utilized as a material to produce biodegradable films and microbe-resistant food preservatives. The presence of positively charged amine groups in the polymer structure has antibacterial properties. Chitosan aids in the preservation of fruits and vegetables by acting as a barrier to moisture, O_2_, and CO_2_ [9]. Chitosan and oligochitosan can suppress the rotting of ginger rhizomes during storage because they can increase the activity of defence enzymes in ginger [5]. When applied to plants, chitosan exhibits three activities: (i) elicitation of host defences, (ii) antimicrobial action, and (iii) formation of films on the treated surface [10].

Another potential coating material is beeswax. Beeswax coating can maintain the moisture content and quality of rhizomes. Waxes are esters of long-chain unsaturated fatty acids with long-chain monohydrate alcohols or sterols [11]. The beeswax coating study, which included a combination of 4% beeswax coating with the addition of paclobutrazol (1,500 ppm), was able to suppress a fairly large percentage of white ginger rhizome sprouts when stored at a temperature of 20–22°C for three months [12]. The different storage facilities have reportedly impacted physicochemical properties such as pH, total soluble solid (TSS), total solid (TS), acidity, and water activity [3].

Polysaccharides and proteins are outstanding materials to form coatings and films, displaying extraordinary mechanical properties. However, they have a limited barrier against moisture transfer. This limitation does not apply to lipids with high melting points, such as beeswax and carnauba wax [13]. Lipid-based compounds have been widely used as coating formers that effectively reduce loss of water and fruit surface erosion, enhance mechanical rigidity, and control inner gas composition [14].

Coatings can be formulated using either a single compound or a combination of compounds using the layer-by-layer method, producing a multilayer coating with high reliability under varied environmental stresses [15]. A bilayer coating that combines chitosan with carnauba wax has been tested on strawberries and demonstrated positive effects in conserving firmness and reducing weight loss [9]. Other research suggested that coating blueberry fruits with chitosan and aloe vera effectively reduced mold contamination and water loss compared to uncoated fruits [16].

Storage is an important stage in postharvest ginger, as proper storage ensures that its quality is maintained for a long period of time. Cold storage is recommended as it significantly extends the shelf life of the commodity. The optimal storage conditions for fresh ginger are 13–15°C with a relative humidity of 90–95% [17]. Casimero et al. [18] suggest that the appropriate temperature and relative humidity for storing fresh ginger are 12°C and RH 75%. Therefore, the availability of cold storage facilities is essential to preserve the quality of ginger. However, the implementation of cold storage is prohibitively expensive for most ginger producers, particularly small-scale farmers, due to the required postharvest infrastructure and facilities. Consequently, this research proposes the utilization of moderate temperatures (around 18°C) achievable through more affordable air conditioning (AC), providing a feasible alternative compared to cold storage. Fresh ginger will be negatively affected when the storage temperature exceeds 18°C, as the germination rate increases.

To date, no information has been found regarding the effect of the double layer coating of chitosan and beeswax on the physicochemical properties of ginger during storage, namely, the moisture and phenolic content, weight loss, TSS, and surface morphology. This study aims to evaluate the physicochemical properties of the ginger bilayer coated with chitosan and beeswax during storage at different temperatures (18 and 25°C) for up to 6 months.

## 2. Materials and Methods

### 2.1. Preparation of Coating Solution

The coating solution consisted of chitosan solution and beeswax emulsion. A chitosan solution was prepared by referring to the method developed by Liu et al. [5]. Chitosan was dissolved in HCl solution (1%) at concentrations of 1.5 and 3%. The pH of the solution was adjusted to 5.5 with the NaOH solution.

Beeswax emulsion was prepared according to the method developed by Yuliani et al. [19]. Beeswax was melted at 95 ± 5°C and then mixed with Tween 80 at a ratio of 1 : 1. Oleic acid and triethanolamine (each at a percentage of 50% of beeswax) were added while stirred with a magnetic stirrer. The resulting solution was added to the hot distilled water (95 ± 5°C) while mixing with an ultra-turrax homogenizer at a speed of 11,000 rpm to form an emulsion solution.

### 2.2. Application of Coating Solution

The coating solution application was performed by immersion and spraying. The ginger rhizome was dipped into chitosan solution (A; A1: 1.5% and A2: 3%) for 10 minutes and then air-dried (25 ± 2°C, RH 82% ± 1%). The second layer of coating was applied by spraying the beeswax emulsion (B; B1: 3% and B2: 6%) on the surface of the air-dried chitosan-coated ginger rhizomes. The double-coated ginger was then air-dried with the help of a fan overnight. The air-dried double-coated ginger (500 g) was packed in perforated polyethylene plastic bags (diameters of 2-3 mm) and stored at room temperature (25 ± 2°C, RH 82% ± 1%) and in an air-conditioned room (18°C). Ginger without coating or packaging was also stored as a control sample.

### 2.3. Physicochemical Property Analysis

#### 2.3.1. Moisture Content

Moisture content was measured using a gravimetric method (drying in an oven). The ginger sample (20 g) was placed into a preweighted aluminium cup. The sample was then dried in an oven (Memmert type UN 55, Germany) at 105°C. Drying was carried out until there was no further weight loss, or around 24 hours. The dried gingers were then cooled in a desiccator for 15 minutes and then weighed. The moisture content was calculated using the following equation [20] and expressed as a percentage on a wet basis (%, w.b.):

(1) $$\begin{array}{c} \text{moisture content }\left(\%,\text{wet basis}\right)=\frac{\text{initial weight}-\text{final weight}}{\text{initial weight}}\times 100\%. \end{array}$$

#### 2.3.2. Total Phenolic Content

The total phenolic content was determined according to the method of Singleton and Rossi with [21] slight modifications. An extract sample and methanol were mixed to create a 500 mg/L solution. After that, 40 *μ*L of the mixture was put into a well plate, and 150 *μ*L of 10% Folin-Ciocalteu was added. Following a 5-minute rest period, 150 *μ*L of 6% Na_2_CO_3_ was added, and it was then left at room temperature for an additional 90 minutes. In this analysis, gallic acid was used as a standard of measurement to calculate the amount of gallic acid equivalent/g of extract (GAE mg/g). With an Elisa Reader Synergy HTX, the absorbance of the extract at a wavelength of 725 nm was determined.

#### 2.3.3. Total Soluble Solid (TSS)

The TSS was measured using the method described by Milosenic et al. [22]. The TSS was measured from extracted ginger juice in triplicate using the Atago DR-A1 digital refractometer (Atago Co. Ltd., Tokyo, Japan) at a temperature of 28 ± 1°C. The TSS was expressed in Brix.

#### 2.3.4. Ginger Peel Surface Structure

The surface structure of the ginger peel was observed using the technique developed by Konarska [23] with slight modifications. First, the ginger peel was dried by a freeze dryer at a temperature of −55°C for 24 hours and sliced into 5 mm × 5 mm in size. Next, the samples were coated with gold (Au) using a sputter coater (Merck Quorum Type Q150R ES, sputter time: 60 seconds, sputter current: 20 mA). Finally, the samples were observed by scanning electron microscopy (SEM) on a Merck Zeiss Type Evoima10 with an electron high tension (EHT) of 16 kV.

#### 2.3.5. Data Analyses

The results were expressed with a one-way analysis of variance at each storage period. The data were reported as the mean ± standard deviation (Sd) of three replications. All statistical analyses were carried out using the SAS Portable 9.13 software.

## 3. Results and Discussion

### 3.1. Moisture Content

**Figure 1:**
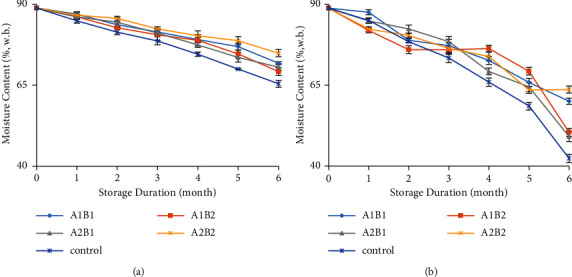
The moisture content at different temperatures ((a) 18°C and (b) 25°C) during storage. A1B1: 1.5% chitosan-3% beeswax; A1B2: 1.5% chitosan-6% beeswax; A2B1: 3% chitosan-3% beeswax; A2B2: 3% chitosan-6% beeswax; control: without coating.

There were changes in the physicochemical properties of coated ginger during storage at different temperatures. The moisture content of ginger showed significant differences, both coated and uncoated, and both stored at 18°C and 25°C (Figure 1). Ginger stored at 18°C had higher moisture contents than those stored at 25°C. After six months of storage, ginger coated with 3% chitosan and 6% beeswax had the highest moisture content, both stored at 18 and 25°C (74.85% and 60.05%, respectively). The coating of ginger can maintain moisture, its natural colour, texture, and aroma [24, 25]. Chitosan coating aids in the preservation of fruits and vegetables by acting as a barrier to moisture, O_2_, and CO_2_ [9].

### 3.2. Weight Loss

**Figure 2:**
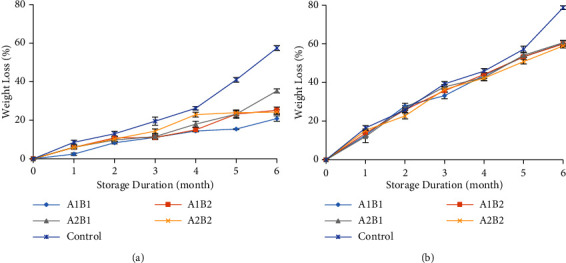
The weight loss content at different temperatures ((a) 18°C and (b) 25°C) during storage. A1B1: 1.5% chitosan-3% beeswax; A1B2: 1.5% chitosan-6% beeswax; A2B1: 3% chitosan-3% beeswax; A2B2: 3% chitosan-6% beeswax; control: without coating.

Ginger stored at a temperature of 25°C lost more weight than that stored at an 18°C temperature (Figure 2). Commonly, during storage, the weight loss increases due to the physiological processes of respiration, transpiration, and other processes by which ethylene gas and some volatile compounds may be lost [26]. The respiration process produces CO_2_ gas, water, and energy [27]. These activities continue during storage and cause the percentage of weight loss to increase. Storage at 18°C resulted in much smaller weight loss than that at 25°C (Figure 2). According to Taghavi et al. [28], the storage of rhizomes at low temperatures may prolong storage and reduce weight loss and sprouting; however, this may result in a higher pathogen incidence than storage at ambient temperature. In addition to the effects of temperature, the results of this study may indicate a protective function of chitosan and beeswax coatings. The chitosan and beeswax coatings cover the stomata on the surface of the ginger, creating a barrier and thus reducing the rate of transpiration and respiration. Foo et al. [29] revealed that chitosan-beeswax coating successfully reduced weight loss and microbial infection while maintaining total soluble solids, fruit firmness, and skin colour of sapodilla fruits. Sultan et al. [30] also obtained similar results on Le Conte pears coated with chitosan-beeswax-pollen grains, which significantly reduced weight loss compared to uncoated samples (control).

### 3.3. Phenolic Content

**Figure 3:**
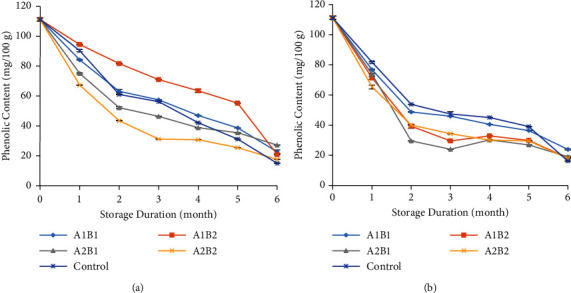
Phenolic content of ginger stored at 18°C (a) and 25°C (b) during storage. A1B1: 1.5% chitosan-3% beeswax; A1B2: 1.5% chitosan-6% beeswax; A2B1: 3% chitosan-3% beeswax; A2B2: 3% chitosan-6% beeswax; control: without coating.

Phenolic compounds are characterised by the nearness of phenolic hydroxyl, which is included in polyphenols, which are an imperative portion of bioactive compounds [31]. Phenolic compounds in ginger include gingerol and its derivatives, which have antioxidant activities [1, 32]. The presence of phenolic compounds is usually used against pathogen attacks or in response to stressful environments [33]. The phenolic content tended to decrease during storage at all temperatures (Figure 3). The phenolic content of ginger stored at 18°C was higher than that stored at 25°C. This was different from that observed in ginger coated with konjac glucomannan, in which the total phenols decreased during the first 3 days and then slightly increased at the end of storage for the 15-day storage period [8]. A similar trend was found in the total phenols of the uncoated ginger, which tended to be higher than that in the coated one for the entire storage period, especially at the ambient temperature. After 6 months of storage at 18°C, ginger coated with 3% chitosan and 6% beeswax had the highest phenolic content (27.56 mg/100 g). The coating could delay the release of bound phenolic during storage, resulting in a higher phenolic content over time [34]. The phenolic content ranged from 14.99 mg/100 g to 27.56 mg/100 g and 17.56 mg/100 g to 23.85 mg/100 g of ginger stored at 18°C and 25°C, respectively.

### 3.4. Total Soluble Solid (TSS)

**Figure 4:**
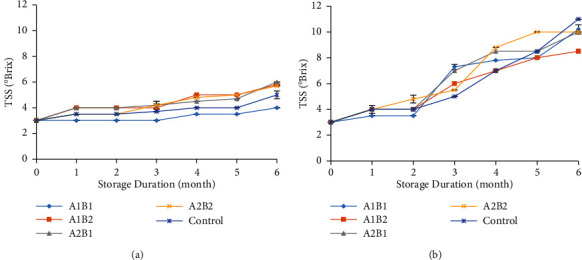
The TSS at 18°C (a) and 25°C (b) during storage. A1B1: 1.5% chitosan-3% beeswax; A1B2: 1.5% chitosan-6% beeswax; A2B1: 3% chitosan-3% beeswax; A2B2: 3% chitosan-6% beeswax; control: without coating.

The total soluble solids (TSS) tended to fluctuate during storage (Figure 4). The decrease in TSS during storage was caused by the levels of simple sugars, which changed to alcohol, aldehydes, and amino acids [35]. The phenolic compounds also contain organic acids, amino acids, and pectin besides sugar [36]. The temperature was one of the factors that affected the increase in TSS. Ginger stored at 18°C had significantly higher TSS than the other stored gingers in the fifth week of storage [18]. The increase in TSS might also be associated with a reduction in product moisture content during storage time [37]. Ginger stored at 25°C showed higher values of TSS than those stored at 18°C. Furthermore, after 6 months of storage, the TSS ranged between 4 and 6°Brix at 18°C. Then at 25°C temperature, TSS was found 8.5 to 11°Brix. The hydrolysis process of polysaccharides into monosaccharides and oligosaccharides will increase the TSS content [38]. The coating treatment of 3% chitosan and 6% beeswax in fresh ginger delays the decrease of TSS during storage at 18°C. It shows that this treatment delays amino acid and antioxidant degradation.

### 3.5. Ginger Peel Surface Structure

**Figure 5:**
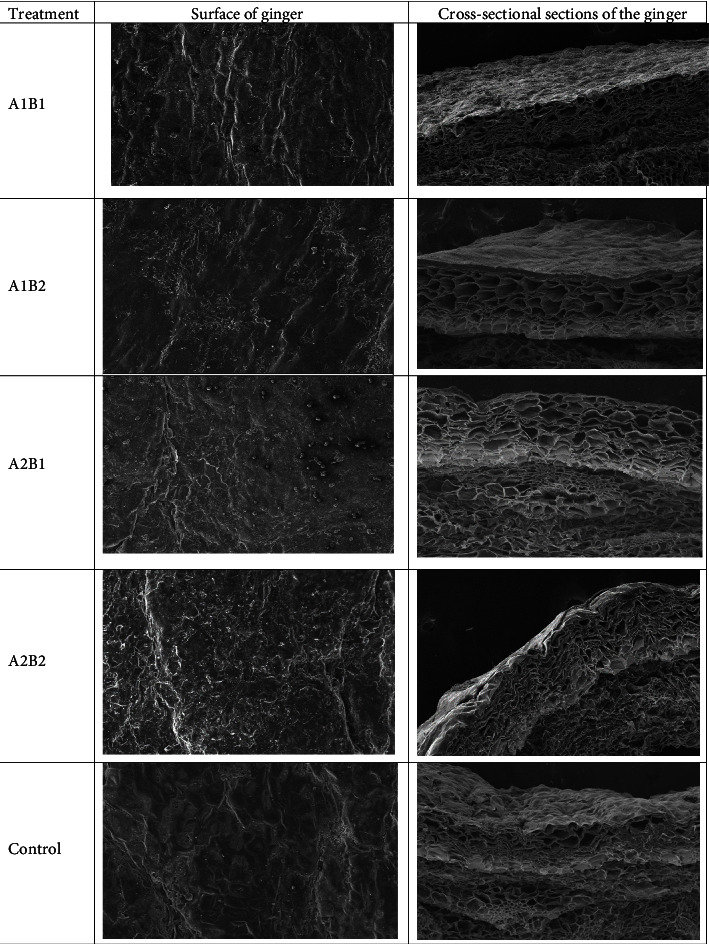
Images of scanning electron microscopes (SEM) of ginger peel, both surface and sectional sections, with a magnification of 100x.

The morphology of ginger peel, both coated and uncoated, exhibited a similar structure (Figure 5). The surface of the ginger showed that the surface of the material is covered by cell membranes and hollow. The cell compartment size of ginger peel coated with 3% chitosan and 6% beeswax was narrower than that of other treatments (Figure 5). The increase of cell wall-degrading enzymes will cause the formation of compartment spaces. This showed that polysaccharide modifications in the middle lamella and cell walls led to cell wall damage and cell separation, resulting in the formation of large compartment spaces [39]. Thus, the coating with 3% chitosan and 6% beeswax might delay cell damage and cell separation, which accelerate water evaporation through transpiration.

## 4. Conclusion

This study confirmed that ginger stored for up to 6 months at different temperatures (18°C and 25°C) experienced decreases in physicochemical properties, and both coated and uncoated ginger stored at 25°C showed the lowest shelf life than those stored at 18°C as indicated by their higher weight loss and reduced visual quality. The coating of ginger with 3% chitosan and 6% beeswax could maintain the moisture and phenolic content, delay weight loss, and delay the increase in TSS and cell compartment size for 6 months of storage. Furthermore, these results indicate the industrial application potential of the coating treatment and storage temperature conditions in maintaining the quality of ginger during long-term storage. Future research can expand our understanding of the effects of coating on broader ginger properties and explore various coating formulations and application techniques to enhance their effectiveness in extending the shelf life of ginger and other products.

## Data Availability

The data used to support the findings of the study are included in the article.
